# Abstracts and Gleanings

**Published:** 1879-04-20

**Authors:** 


### ABSTRACTS AND GLEANINGS.

**Post-Mortem Delivery.**—Dr. Edward L. Duer, of Philadelphia, has collected 55 cases of removal of a living child after the death of the mother, which he tabulates with his views and conclusions in a valuable paper in the American Journal of Obstetries. This is by no means a speculative inquiry. Every practitioner of obstetrics is liable to have a dead, undelivered mother before him, and the possibility of the delivery and rescue of the fœtus should be so clearly established that there should be no hesitation or delay in his procedure. Let us, then, briefly follow the points of Dr. Duer in his admirable resume :

1. The period of pregnancy, when the child may be delivered with any favorable prospect. "For all purposes of practice, it may be assumed that an infant, delivered by Cæsarean section, is as likely to live as one born at the same period of pregnancy in any other way; and as there is incontestable evidence of children having lived who were delivered from a living mother, at the sixth month of utero-gestation, and especially as we are dealing entirely with possibilities and not probabilities, we will be safe in the adoption of a rule : To perform forcible dilatation, or the Cæsarean section, on all otherwise favorable cases, which have attained to the neighborhood of the sixth month of pregnancy."

2. The length of time after the death of the mother, when operation promises success; of course, the sooner after the death of the mother the child is delivered the greater the possibility of good results. But, "if a child is manifestly living, as indicated by its movements, or by the sound of the fetal heart, there can be no question of the propriety of an operation; the converse of this, however, is by no means true, and we are obliged, then, to fall back on observation and recorded experience."

"In the Hunterian Society discussion, Dr. Lever relates that he has several times seen the movements of the fœtus in utero, half an hour after the mother's death." While in the cases collected by Dr. Duer there were 13 delivered between 10 and 15 minutes; two cases after one hour; two cases after two hours; there are cases reported of living children being removed after a much longer interval of death than any of these, but they are not regarded as reliable; as, for example, that of the Princesse de Schwartzenberg, where the infant was removed twenty-four hours after the death of the mother and ultimately survived. Even this case is recorded as "well known" and "still believed to be true."

One of the most important, for practical consideration, of the cases collected by Dr. Duer, is that reported last year to the Cincinnati Obstetrical Society, and printed in the Lancet and Clinic, by that careful and reliable practitioner, Dr. J. L. Cleveland. Indeed, Dr. Duer speaks of Dr. Cleveland's case as "in all respects the most interesting of the series." "The mother is represented to have had convulsions (probably uremic) for about two weeks, and is supposed to have died in one. For reasons mentioned, the time which elapsed before the operation was 'full one hour.' The child was removed asphyxiated, but the heart-beat was perceptible. It gasped in a short time, and in the course of an hour was fully restored. It was small, near full term, and is still alive and in good health.—*Obs. Gazette.*

**Remedies for Dilating the Cervix During and Preparatory to Labor.**—Dr. Sell related a case as an example of several, in which he had used the concentrated tincture of caulophyllum, or squaw weed, with the happiest results, as a remedy to ward off tedious labor. The remedy was especially applicable in those cases in which the woman had habitually suffered severely during the first stage of labor. As a preparatory remedy in such cases it should be administered in twenty-drop doses three times a day, for three or four weeks previous to confinement.

Dr. Merrill remarked that he had witnessed similar results from the use of castor oil during labor. He referred to cases in which he had found the os rigid, had ordered a dose of the oil, and, by the time the bowels were freely evacuated, the rigidity had disappeared, and speedy delivery was effected.

He further remarked that he had used castor oil with good effect in cases in which the uterine contractions were weak and the os was considerably dilated. Given in half-teaspoonful doses every ten or fifteen minutes, the oil had produced marked uterine contraction as rapidly as he had ever obtained by the use of ergot.

Dr. Sell regarded the oil as a dangerous medicine to be given during the latter months of pregnancy, for if it had the power to excite uterine contraction, it might produce premature delivery.

He also referred to gelseminum as a valuable remedy in cases of rigid os during labor.

Dr. Merrill remarked that he never ordered castor oil, except when he was convinced that it was time for labor, or the process had already commenced. He always charged his patients not to take castor oil during the last months of pregnancy, if remedies were needed to keep the bowels open, because of the liability to excite uterine contraction.

Dr. F. V. White remarked that when he was an interne in Bellevue Hospital, it was customary to administer castor oil to the lying-in women on Sundays, and there were marked results following its administration.

He also asked Dr. Sell if he had not obtained as satisfactory results from the use of chloroform in cases of rigid os during labor, as from the use of gelseminum.

Dr. Sell replied that he preferred gelseminum to chloroform.—*N. Y. Acad. of Med.—Med. Rep.*

**The Treatment of Alcoholism.**—F. P. Atkinson, M.D., in London Practitioner, says:

Some of the most distressing cases we, as medical men, are called upon to attend are those of alcoholism, and it has unfortunately fallen to my lot during the last few years to have several from time to time under my charge. A good deal has been written by different persons with regard to treatment, but I do not think this ought to deter one from putting on record his own personal observations, since it is only by accumulation of evidence that proper conclusions can be arrived at.

As far as I can see, there would appear to be three different stages in the disease, viz :

1. Sleepiness, accompanied by a hard quick pulse; loss of appetite in the morning, and morning sickness.

2. Drowsiness, accompanied by a slow, somewhat compressible and excitable pulse; complete loss of appetite,, and constant sickness. The blood has in it an excessive amount of hydrocarbon.

3. Delirium, accompanied by complete absence of sleep and the presence of horrible apparitions, especially at night. The pulse is small, quick, easily excitable and compressible. The blood is deficient in red corpuscles. Hydrocarbons are present in poisonous quantities; the brain undergoes little or no repair. The vaso-motor nerve influence is almost entirely lost.

The treatment I have found beneficial in each stage is the following:

First stage. Tinc. rhei, m x; tinc. card. co. ʒss; acid hydrocyanic, dil., miij; sp. chloroformi, mxv; tinc. hyosciami aquæ ad. ℥i, quartis horis.

Second stage. The treatment should be the same as just described, only it is as well to omit the prussic acid, as there is not the same excitement present.

Third stage, Chloral should be given in thirty-grain doses every four hours, till sleep comes on, and then repeated as often as necessary. The nourishment should be by no means forgotten, and stimulants should be strictly forbidden.

If chloral is gone on with beyond a certain time, a sleepless condition recurs, when nux vomica and gentian should be given as follows: tiuc. nucis vomicæ, m x; tinc. gentian co., ʒss; ess. limonis, mi; sp. chloroformi, mxv; aquæ ad. ℥i. ter quaterve die. This rarely fails to reinduce sleep, but if persisted in long after it has produced its effect, sleeplessness returns. When this is the case the tincture of gentian, calumba, or chiretta should be given alone.

These remarks I am afraid, will have but little interest for the scienfic observer, but they cannot, I think, fail to be of use to those engaged in general practice.

To show to what extent alcohol may be present in the blood, I may mention I have known it to burst into flame on a light being applied to a cut head, and Prof. Ogston, of Aberdeen, has told me he has been able to set it alight, when he has cut open the bladder of a man who had died in a fit of alcoholism.

**Pathology of Hysterical Cough.**—Dr. W. S. Dodd, in Medical and Surgical Reporter, says:

*Case* 1. Mrs. J. S., aged thirty-four, hysterical, suffering from uterine polypus, was taken with violent cough, and for ten days I could get nothing to alleviate it a particle; finally, in examining the spine, I discovered that pressing on the fourth and fifth dorsal vertebra increased the cough. I ordered a blister to the spot, which remained twelve hours, and the cough vanished completely. I subsequently got rid of the polypus, and the patient has since borne a thirteen and a half pound child, and has got good health.

*Case* 2. Mrs. S. A. T., aged twenty-two, hysterical. I had procured an abortion with her, for relief of puerperal convulsions, on January 24th, 1877. She remained in delicate health, but became enceinte in August. I was called October 1st, and found her emaciated; had retained no food for a week, and in the last two days retained nothing, not even a spoonful of water; the spine was excessively tender, and pressure on the lower dorsal vertebra caused retching. Ordered bis. sub. nit. et ingluvin, equal parts, five grains every three hours, and a blister to the spine. She vomited everything until the blister had done its work; then she retained the medicine, and in a short time was able to take a light diet. October 3d. Some cough; retains food. October 4th. Cough violent, and continued so for three days, interfering with the digestion. October 8th. Find some vomiting; cough still violent, and excited by pressure on the dorsal vertebra; applied blister to the spine, and it remained twenty-four hours without blistering, over the third, fourth and fifth dorsal vertebra. October 9th. Still coughing; applied a fresh blister where the other failed, and in ten hours it blistered, and in twenty-four hours the cough was gone. She recovered, and in May, 1878, was delivered ot a fine child, at full term.

*Case* 3. Mrs. A. has uterine trouble of long standing. Was called November 17th, 1878, and found she had diphtheria, which ran a severe course. The fifth day of the disease had slight cough; was fearful of extension of disease into the larynx, but all remedies for it failed to even check its progress. On the decline of the diphtheria, in the third week, the cough still increasing, I examined the spine and found that there was cough on pressing the dorsal vertebra; applied the blister, and when it produced sufficient counter-irritation the cough subsided.

My conclusions are, that a nervous or hysterical cough comes originally from genital disturbances, through the spine, and my treatment is satisfactory to me and my patients.

**Arrest of Milk Abscess.**—W. P. Swain, F.R. C. S., reports as follows to *London Lancet:*

The following case, although by no means without precedent, is so marked as to deserve record.

Mrs. W——, a primipara, was confined on July 13th. The labor was natural, but the child very large. The forceps were used in the last stage. She progressed favorably up to July 26th, but at one o'clock in the morning of that day she had a shiver, followed by burning heat, with fullness, pain, and great tenderness on pressure in the right breast. The breast had been somewhat troublesome to nurse with throughout. I saw the patient at 1 p. m. She was then flushed and anxious, with a pulse 120 and a temperature 103.6°. The breast was very full, tense and painful, especially toward the axilla, where a spot much harder than the surrounding tissues could be felt. The following treatment was prescribed: An immediate dose of ten grains of quinine; two-drop doses of tincture of aconite in a drachm of water every ten minutes for four hours, and then every hour; and the extract of belladonna softened with a little glycerine to be smeared over the breast. The breast pump was also ordered to be used. I saw her again at 10 p. m. The treatment had been faithfully carried out, except that the dose of quinine had not been admistered until 7 p. m., owing to the chemist being reluctant to dispense so large a dose of quinine without special authority from me. I found the breast much less tender, the pulse 88, and the temperature 101°. Five grains of quinine were ordered to be taken every four hours, and the aconite to be continued every hour. The next day (July 27) I found the patient more comfortable. The child had taken the breast, which was hardly now at all tender, the hard spot having vanished. The pulse was 80, and the temperature 96.6°. From that time she progressed favorably, without further bad symptoms.—*Obs. Gazette.*

[Two drops of aconite every ten minutes for four hours seems excessive.—Ed. Rec.]

**Prussian Blue in Malarial Fever.**—Through the suggestion of a friend of mine, wrote Dr. W. L. Martin, I was led to the use of the above article in some stubborn cases of intermittent fever, more particularly that form termed "dumb ague or chronic chills." Now, after the use of it for several years, I have found it an efficient remedy, and have rarely been disappointed in being able to effect a cure. It must be given in pretty large doses, much larger than is directed in our books, to have this desirable effect, say ten grains three times a day. This amount, in my hands, has proved sufficient for an adult. In looking at the article chemically, we might fear using such large doses, but, after experience, I have seen nothing bad result from it whatever. Given in powder, dropped upon the tongue and washed down with a little water, is the most eligible way of administering it. The taste is somewhat similar to that of powdered charcoal, and but few complain of it being unpleasant to take; in this way even children take it readily. Given in pilular form, a dose would make three pretty large pills; this amount having to be taken three times a day, we would find but few that would submit to it. Prussian blue being a chalybeate has the effect of that class of remedies, as well as that of an antiperiodic, and I found it to be most efficient in those cases where there is a "run down" or anæmic condition, and when a cure is effected it is more permanent than that from quinia. There are some cases where quinia will not make a permanent cure, even when given under the most approved plan, which, I believe, is after the chill has been arrested by it, to keep them from returning by giving a full dose the day preceding the seventh, fourteenth and twenty-first day following the last chill. Now, in these cases, where quinine has so failed, the ferrocyanide of iron comes in as just the thing.—*Med. and Surg. Rep.*

**A Simple Aspirator.**—Dr. G. A. Harman, Lancaster, Ohio, writes to the Clinic: As every physician now wants an aspirator, and as simplicity, convenience, durability, reliability, and price are all desirable and necessary considerations, with your permission I will describe one answering all those requirements.

Take a rubber tube, about one-half or one-third of an inch in diameter, about three or four feet long, sufficiently elastic to allow of compression, but not much stretching, and enough resiliency to return to its shape when allowed. The tube used in the construction of the Davidson syringe nearly answers the purpose; but it is rather hard to compress, and stretches too much. A lighter tube, with a longitudinal linen fiber, would suit the purpose better.

Having a proper tube, you stretch one end over the head of an aspirating needle. You now have your aspirator ready for use.

Thrust the needle into the cavity to be aspirated; then grasp the tube with the thumb and finger of one hand, as near the needle as possible. with sufficient tightness to completely obliterate the hollow, and so, with thumb and finger of the other hand close up to first, holding tightly with proximal hand (in relation to needle), slide the other down the tube, keeping the hollow all the time obliterated, thus exhausting the tube of air. When you have slid down the tube a sufficient distance, say a foot or more, you have between the fingers of the two hands a vacuum, which, upon releasing the grasp of the proximal hand, is filled by the rushing in of fluid from the aspirated cavity. Drop the distal end of the tube, and you have a syphon which will completely empty the aspirated cavity. Should the tube be too elastic, it will be found convenient to follow up the distal hand by letting go with the proximal, and taking a new hold near the distal, while holding the tube firmly with the latter; thus compressing a few inches of the tube at a time; or the tube might be very easily and completely exhausted by winding it tightly around a smooth, conical stick, beginning close to the needle, then securing the distal end and pushing it off.

Should it be desirable to immediately see what kind of fluid is being drawn away, the tube can be divided near the needle, and a short glass tube inserted in the section.

This little device (for which I claim originality) will enable every doctor to supply himself with an aspirator at a cost of only about a dollar.

**Hypodermic Injection of Brandy.**—Within the last few weeks I have had a case in my practice here in which the subcutaneous injection of brandy undoubtedly prevented a fatal issue. Having had occasion to use the forceps for the removal of a child that had unfortunately died *in utero*, under circumstances which precluded the use of chloroform and necessitated the loss of a considerable amount of blood, I had no sooner secured a proper contraction of the womb than the woman sank into a state of collapse so profound that I could scarcely determine whether she was really dead or alive. There was no pulsation in the radial artery, the heart seemed motionless, the respiration ceased, the extremities became as cold as ice, the jaws were absolutely rigid, and the face assumed the very aspect of death. Instantly lowering her head, I injected two drachms of pure brandy over the apex of the heart, and resorted to artificial respiration, while the nurse under my orders made a liberal use of warmth and revulsives. In response to these means there came a slight flutter about the heart and a single feeble sigh, and then a lapse to the same awful state of silence and inaction. Repeating the injection of brandy and persisting in the other measures, I succeeded after some moments in securing a somewhat more decided movement of the heart and a rather stronger respiratory effort, followed by another speedy return to the deathlike condition which I have already described. And so, with alternations of resuscitation and relapse, amid gleams of hope and intervals of despair, I labored on for more than two hours before the slightest continuity in the cardiac and pulmonary functions was established, and it was only after the lapse of a much longer period that a sufficient amount of reaction developed itself to enable me to decide whether death or life was to be the issue of the struggle. Eventually, however, success crowned my efforts; the state of collapse passed away, and, after a long and weary subsequent illness, the patient lives to unite with me in blessing the days on which brandy and hypodermic syringe were invented; for they restored her to life and relieved me from one of the most painful and trying positions of my whole professional career.—*North Carolina Medical Journal.*

**The Plague.**—The Bulletin of the Marine Hospital Service states that official reports of European medical officers in China show conclusively that true "bubonic plague" has prevailed extensively in that empire during the thirty years preceding 1873, when it was supposed to be wholly extinct. The reports also show that owing to the meagre facilities for communications with Central Asia, virulent epidemics may ravage extensive districts of that country without any knowledge of their existence extending to Europe. The reports present records of the disease having prevailed in the Province of Yunnan, to which it seems to have been introduced from Burmah, during twenty of the thirty years in question, varying in intensity in different parts of the province, and in different years.

From facts, which have been obtained from official sources, and are in the main well attested, it seems proper to conclude that instead of the late outbreak being due to the spontaneous regeneration of the virus of the plague in the valley of the Volga, or, at the furthest, in Persia, the disease was reintroduced from China into Persia, and thence to Russia, local conditions in each instance probably favoring its development. Of these conditions no authentic account will be obtained until the international Commission of Experts, who are visiting the infected district, make their report. The return of cold weather, combined with the stringent measures adopted by the government, seem to have confined the late violent outbreak to the limited district where it first appeared. The American ministers to Austria and to Russia report that the disease has manifested such an extremely virulent and contagious character that great alarm exists in the whole of Eastern Europe, and urge upon the government the necesiity of taking measures to prevent the possibility of the introduction of the disease into the United States. The measures already taken by this government for preventing the importation of goods from the infected districts, except under proper precautions, are, for the present, considered sufficient for this purpose, especially if the ports of entry are kept free from the unsanitary conditions that favor the spread of epidemic disease.—*Med. aud Surg. Reporter.*

**Marriage and the Microscope.**—Dr. C. Heitzman, of New York (N. Y. County Medical Society, Dec. 23, 1878) read a paper in which he claims to be able to determine the constitution of an individual by means of microscopical examination of the blood, without knowing anything of his former life. The better the constitution, the fewer are the colorless blood corpuscles. These corpuscles are partly homogeneous, or at least coarsely granular in strong men; they are also paler in color (yellow) in weak persons. He says:

"In fact, the microscope reveals so much of the general health of a person, that more can be told by it in many instances than by naked eye, or by physical examination, as well as on percussion and auscultation. Marriages should be allowed in doubtful cases, only upon the permit of a reliable microscopist. Last season a young physician asked me whether I believed in marriage among kindred? He fell in love with his cousin, and so did the cousin with him. I examined his blood and told him that he was a nervous man, passes sleepless nights, and has a moderately good constitution. The condition being suspected in the kindred lady, marriage was not advisable for fear that the offspring might degenerate. So great was his faith in my assertions, that he gave up the idea of marrying his cousin; offering her the last chance, viz: the examination of her blood. This beautiful girl came to my laboratory, and very much to my surprise, I found upon examination of her blood a first-class constitution. The next day I told the gentleman: 'You had better marry her.'"—*N. Y. Med. Record.*

**Rheumatism.**—Dr. Richard said he had used salicin for acute articular rheumatism, in five to ten grs. doses, without benefit.

Dr. Snow has used salicylic acid in a number of cases of acute rheumatism with the most favorable results. In one case he gave it in doses of grs. x ad. xv. The pain subsided, but the head symptoms—similar to those induced by large doses of quinine—were so severe that the medicine was discontinued, when the pain again returned. Similar doses stopped the pain, but caused the head symptoms as before, when smaller doses completed the cure without further trouble.

Dr. Borrowman has given the acid in doses of grs. v ad grs. xx, every two hours, without observable effect.

Dr. Kaiser usually gave five grs. each of salicin and potass. nit. dissolved in glycerine. Tinct. aconite three parts and tinct. opium one part, is an excellent local application.

Dr. Leonard. Salicylic acid may be good in malarial cases.

Dr. Lyster favors the warm room, and does not think it increases the temperature of the patient suffering from rheumatism. He generally uses the following:

| | | |
|---|---|---|
| ℞ | Tr. colchici sem. | ʒ v |
| | Potass. iodidi | ℥ ss |
| | Tr. quassiæ | ℥ iijss |
| | Syr. sarsaparillæ | ℥ iv |

M. Ft. sol. S. Two teaspoonfuls.

Gave morphine to ease pain, and used locally cloths out of hot alkaline water, covered with oiled silk, or simply envelope the part in cotton batting.

Dr. Rouse prefers quinine to salicin in malarial districts,—*Proceedings of the Wayne County Medical Society.—New Prep.*

**Ergotine in Acute Ophthalmia.**—Dr. Planat, of Nice, states, in the *Journal de Therapeutique*, that he has found ergotine act with efficacy and promptitude in proportion as oculo-palpebral phlegmasiæ are simply inflammatory. In blepharo-conjunctivitis the improvement is first observed in the conjunctiva; and in keratitis, although still very active, it is a degree less so than in the more superficial affections. It is also of great service in iritis, rapidly subduing the acute manifestations and preventing their extension to the external membranes of the eye. When these last are the seat of a chronic fluxion dependent on a scrofulous or dartrous diathesis, ergotine, without influencing the constitutional affection, acts none the less efficiently on the inflammatory element—a fact of importance, as by generally preserving the eye from plastic deposits, corneal ulcers, and consecutive staphylomas, it allows of the treatment for the diathesis being more promptly put into force. The formula which Dr. Planat recommends is from one to one and a half gramme of ergotine in twenty of glycerine or rose water, of which from eight to ten drops are to be inserted in the eye every two hours. Where there is violent inflammation of the eyelids or distention of the conjunctiva, a rag wetted in this mixture should be left on the parts for some hours. In general, two or three days suffice for the subdual of the most intense blepharo-conjunctivitis. Dr. Planat has employed the ergotine in this way, with invariable success, for several years past.—*Med. and Surg. Rep.*

**The Treatment of Acute Rheumatism.**—Prof. Stille, in the Journal of Materia Medica, says:

The treatment of simple acute articular rheumatism may be abandoned to palliatives and nature. Apart from complications, such cases nearly always get well under rest and good nursing. Try and disabuse yourselves of the idea that their cure is dependent upon medicines alone; to help nature is often the best we can do. No treatment was ever invented which stopped a case of acute articular rheumatism. It cannot be accomplished by bleeding or sweating or purging, by niter, by tartar emetic, by guaiacum, by alkalies, by salines, by salycilic acid, or by anything else. The physician can palliate pain and perhaps shorten the attack; can perhaps prevent or control complications and stiffness in the joints, but he can not arrest the disease. Where rest, proper diet, and warmth are enjoyed, most cases will get well just as soon without as with the use of other remedies. Dr. Austin Flint, of New York, in support of this statement, subjected some patients, a number of years ago, to the expectant treatment, and found that they made just as rapid and just as complete recoveries as those cases under active medication.

**Fistula in Ano.**—During the last five years I have had under my care several cases of fistula in ano. All but the last were subjected to the division of the sphincter ani with the knife, and although varying considerably in the length of time required to heal the wound, made good and perfect recovery. With the last I adopted a different method. It was a complete fistula, having one opening in the bowel above the sphincter, and another (external) near the anus, whence frequently fecal matter exuded. Having cleared the bowels thoroughly, and then secured them against disturbance for two or three days, I introduced to the bottom of the fistula, in a hollow tube, some pure carbolic acid in a liquid state. The first application seemed to close the internal opening; the second, introduced the same way, required a less deep insertion; the third less still, and so on until the sinus became completely closed. About two months have elapsed. The patient has had no return of the affection, and declares himself perfectly well.—*Cor. London Lancet.*

**Hyoscyamine.**—A correspondent in the Lancet admonishes his readers to order "Merk's Extractive Hyoscyamine" in order to obtain uniform results from the remedy. The dose is that of atropia (which it resembles in physiological action) gr. one-eightieth to one-sixtieth.

**Berlin Treatment of Scarlet Fever.**—In the last volume of the reports of the Charity Hospital, Berlin, Professor Henoch has an article on scarlet fever, in which he treats of the anomalies of its progress, its malignancy, complications and treatment. On the latter point he recommends, when stimulants are indicated, to employ cognac, coffee, camphor and musk. When swallowing is interfered with by the swelling of the fauces, he uses nutritive enemata and local hypodermic injections of camphor.

℞ Camphoræ pulv........................ 0.6 G
Alcoholis
Aquæ distillat........................aa 5.0 G. M.

Inject a syringeful.

When the pyrexia is marked, he employs lukewarm baths; cold baths he considers dangerous, as leading to collapse; but he is not afraid of cold sponging and the hydropathic pack.

**Fatal Tetanus Following Ligation of Hemorrhoids.**—For four days the patient complained of a little soreness but otherwise was apparently comfortable. The tissues included in the ligature became sloughy in appearance and diminished in size. About noon on the 14th, the patient complained of stiffness of the jaw; he seemed restless and nervous; his pulse and temperature remained normal. The ligature came away. The rigidity of the muscles of the jaw gradually increased so that on the following day, the patient was unable to open the mouth at all. The muscles of the back of the neck were also affected so that the head was drawn backward; still the breathing was regular and the temperature normal, though the pulse was slightly accelerated. At 11 o'clock on the night of Jan. 15th, he had a convulsive attack, lasting about five minutes and attended with great difficulty of respiration. This was succeeded by four others, each following its predecessor at a shorter interval and being more severe, and in the last he died, at 5 A.M., January 16th, the immediate cause of death being asphyxia. No autopsy could be obtained.—*Hospital Gazette.*

**The Croton Oil Treatment of Nævus.**—An instance of it is given by Dr. H. F. Sigier, in the Michigan Medical News. He writes: "The tumor was situated in the centre of the left cheek, and in size about as large as a dime. I procured a cork the size of the tumor, into which I inserted several fine needles, letting the points project ⅛ of an inch. I then immersed the points of the needles in pure croton oil, and plunged them into the tumor. A little swelling followed, and several vesicles formed soon after. The second day a crust formed over the whole tumor. This was repeated three times, at intervals of five days, and no other treatment was required.—*Med. and Surg. Rep.*

**Glycerine as an Enema in Constipation.**—Dr. L. W. Hanson, of Davison's Station, Mich., writes to the Medical Record, that for the past two years he has been in the habit of using glycerine in all classes of cases where he desired to produce evacuation of the bowels by enema, whether of acute disease or habitual constipation. He uses it one part glycerine to six, eight or ten parts of tepid water, and finds it to act more pleasantly and efficiently than any other agent ever used for the purpose.

**Is Urticaria Caused by Sulphate of Cinchonidia?**—Dr. Kemper, of Muncie, Ind., states that he and his partner have frequently remarked upon the unusual number of cases of urticaria which have occurred this season, especially among children attacked with malarial fevers. During this time they have used the sulphate of cinchonidia almost wholly in the place of quinine; and families keep and use the former. By such persons they have been quite commonly consulted for "hives" and "nettle-rash," which occur early in some cases, later in others. In many instances, instead of the ordinary form there was marked puffiness of the face, eyelids, and sometimes of the extremities. In one case the eyes were nearly closed by the swollen lids. The cinchonidia was left off, and the swelling disappeared. Two weeks afterwards, when a few doses had been taken, the swelling reappeared. Dr. K. states that to a patient in whom quinine had several times produced an erythema of marked degree, he gave six grains of the cinchonidia. At his next visit she, with a reddened face, accused him of having given her quinine again. She gradually became completely covered with an erythematous blush, and subsequently underwent general desquamation. He was formerly disposed to believe that malaria caused urticaria, but has now abandoned that idea.—*Cin. Lancet and Clinic.*

**Sulphuric Ether in Lumbago.**—I had one morning a very urgent message to go some miles. At the time I was almost unfit to move in bed from lumbago, and to put my foot to the ground to walk was impossible, from the pain it gave me. I once before had the rheumatism in my arm and shoulder, and when one day working with ether I happened to inhale a small quantity, and to my surprise the rheumatism almost immediately left. Having this in my remembrance I got the ether-bottle and inhaled the ether on the folded tip of my pocket-handkerchief for about twenty minutes, not entirely putting myself under its influence; and in less than an hour I was able to rise with little or no pain, and drive out, make my visit, and a good many other visits besides; and on my return I felt the pain had increased a little; I inhaled some more ether, and the next day I may say I was entirely better. I have also tried it on others with the same miraculous effect in lumbago.—*Dr. David Smith, in Brit. Medical Journal.*

**The Topical Application of Chloral Medicated with Camphor.**—The mixture of chloral and camphor is transformed by heat into a thick oily transparent liquid, resulting from the solution of the camphor in the chloral hydrate, which thus loses its proportion of water. This topical application does not act like chloral by revulsion, for it does not produce the slightest hyperemia of the skin. Its action appears therefore to be due to its absorption. Dr. Sune, who has made out these facts, has seen several cases of pain in the side and slight attacks of neuralgia cured by this new medicine.—*Independencia Medica.*

**Battey's Operation.**—The Paris Medical says of Battey's operation: "It is not only rash, but is to be condemned for its boldness. We cannot approve the conduct of a surgeon who thus plays with the life of his patients, especially when we remember the gravity of deep lesions of the peritoneum. We are happy to state that no French surgeon has followed the footsteps of the American."

**Use of Chloroform in Diseases of the Heart.**—On this subject Mr. Vergely, of Bordeaux (*La France Medicale*), remarks that there is a difference of opinion, some asserting that chloroform is very useful and others that it does harm in affections of the heart. In Mr. Vergely's memoir, to which M. Dieulafoy has recently drawn the attention of the Societe Medicale des Hopitaux, three principal points are established.

1. That the existence of heart disease does not contraindicate the use of anæsthetics.
2. That chloroform is a sedative in this class of diseases.
3. That it should be used with discretion.

In some cases of severe palpitation chloroform may be successfully administered. Also in some cases of dyspnœa and palpitation arising from mitral insufficiency, either alone or conjointly with hypodermic injections of morphia. M. Vergely has also given it without any accident in angina pectoris, and in certain other affections of the heart characterized by dyspnœa and palpitation. From inquiries he has made into the literature of the subject, he concludes that this agent has been employed too timidly and unsystematically.—*Med. Rep.*

**Warm Fomentations to the Head in Cases of Uterine Hemorrhage.**—Dr. Koehler (*Allg. Med. Central-Zeitung*) states that he has, for the last seven years, in cases of uterine hemorrhage, applied warm fomentations to the head, to prevent anæmia of the brain, and also to the heart. Hot sand bags are also very efficient, and the patients often will bear sand which is so hot that it can scarcely be touched with the hand. As soon as the fomentation or bag has been applied, consciousness is restored; the pulse grows stronger; the patient herself states that she feels better, that the ringing in the ears has ceased, and that she likes the appliance. As soon as it becomes cooler, she wishes it to be renewed. Dr. Koehler has, he says, saved patients even in the most dangerous cases of hemorrhage by this proceeding, by which the physician never loses time, as the fomentations may be watched and renewed by any one. This method has been found equally efficient in anæmia caused by epistaxis, hemorrhages produced by wounds, etc.—*Med. Rep.*

**Novel Use for Apomorphia.**—At the late meeting of the French Association for the Advancement of Science, Mr. Verge related the case of a child with a plum-stone empacted in the gullet. No instrument being at hand for the extraction of the foreign body, recourse was had to emetics. Ipecacuanha failing to induce vomiting, it was decided, after consultation, to inject apomorphia subcutaneously. Two injections were practiced, amounting in all to two and a half milligrammes of the alkaloid, not a large amount. Vomiting was soon excited, and the foreign body expelled.—*London Lancet.*

**Herpes Zoster.**—Dr. Walther, of Mittweida, reports the following singular occurrence of zoster. A student affected with the disease removed from his room. The next occupant shortly after was attacked with the same affection. This one also removed, and the third occupant, also a student, was immediately attacked with the same disease. —*Allgem. Med. Central Zeitung.*

**Epilepsy.**—Dr. Hammond, in his work on Diseases of the Nervous System, in detailing the successful treatment of a very obstinate and apparently hopeless case of epilepsy, says:

We placed the patient upon a formula almost identical with Dr. Brown-Sequard's celebrated one for epilepsy, substituting the sodium for the potassium salt, in consequence of its less depressing effect and of its greater tolerance by the system, giving three times daily twenty grains of the former with a half drachm of Squibb's fl. ext. ergot. She began the remedy in October, 1874, and took it faithfully for a year and a half.

Her mother stated that at the four subsequent menstrual periods she had three severe epileptic seizures daily. They then disappeared entirely. Continued medicine, notwithstanding their cessation, for over eighteen months. The fits have never recurred since early in February, 1875, now three years and ten months.

**Purulent Ophthalmia of Infants.**—Iodine is more soluble in distilled water of cherry-laurel than in simple distilled water. Ten drops of tincture of iodine disappear in ten grammes (168 minims) of distilled cherry-laurel water, by a chemical transformation, the mixture being free from coloration; while in the same quantity of simple distilled water it immediately colors it, and gives, in a short time, a precipitate of iodine. Dr. Lutton prepares a liquid containing one gramme (16 minims) of tincture of iodine to twenty grammes (324 minims) of cherry,, or at the 20th. It has the color of pale cognac, and forms a collyrium of unquestionable efficacy in the purulent ophthalmia of newly-born children. By means of a glass dropping tube, this liquid is instilled between the lids four or six times per day, and these are also bathed with it as abundantly as possible. It is greatly superior in efficacy to nitrate of silver, and occasions no inconvenience.—*Union Medicale et Scientifique du Nord-Est.*

**Treatment of Enlarged Glands in Scarlet Fever.**—A writer in a late number of the London Lancet: I have lately adopted a very simple method of preventing the great suffering which little patients sometimes have to undergo from enlarged glands in scarlet fever. The attendant is directed on the slightest indication of enlargement to rub gently into the neck every two hours by day and every four at night linimentum ammoniæ, with a little tincture of opium added; each application to last ten or fifteen minutes; and, to insure the necessary perseverance, warning is given of the probable serious consequences should the enlargement continue. I have recently had a large number of cases of scarlet fever, some of them severe, but I have had no enlarged glands. The friction obviously prevents the collection of morbid material in the gland and subsequent adenitis.—*Med. News.*

**Diphtheria.**—It has been lately suggested that rotten potatoes and apples are causative of diphtheria, the fungi of these being identical with the fungiof the diphtheritic ulcer. An observer says that the disease invariably attacks families in which the Irish tuber is eaten, and that, during epidemics, families who abstained from the vegetable enjoyed immunity from the disease. Sweet potatoes are not supposed to be similarly injurious.

**Treatment of Erysipelas by Injections of Carbolic Acid.**—Dr. Hueter contributes the experience of the Greifswald hospital to this question. A solution of carbolic acid in alcohol and water is injected by the Pravaz syringe into the affected skin, at points sufficiently near to each other to control the inflammation. In many cases two or three injections are sufficient; in severe ones five are generally found to suffice; while in the worst as many as twelve have been used. After making them, the skin is kept covered with a carbolized compress, which is changed two or three times a day. If the erysipelas is accompanied by lymphangitis and lymphadenitis, mercurial ointment is spread thickly along the course of the red streaks and over the swollen glands.—*Berliner Klinischenschrift.*

**Occurrence of Herpes During the Administration of Arsenic.**—Dr. Finlayson, of Glasgow, reports, in confirmation of the opinion expressed by Hutchinson that zoster is frequently developed during the use of arsenic, two cases in which this affection occurred in young women while under the action of the drug. [Observations of such occurrences are not yet numerous enough to warrant the positive conclusion that the alleged connection is more than accidental. Zoster occurs during the use of other drugs. For instance, we have under observation at this time a fully-developed case upon the thorax in a syphilitic patient under treatment by mercurial inunction.—*Rep.*]—*Hospital Gazette.*

**Treatment of Mammary Abscess.**—Dr. A. M. Armstrong writes: "For ten years past I have not had the unpleasant task of opening mammary abscess, where either chloroform, chloroform liniments, or the atomized sulph. ether had been freely applied to the affected breast, before suppuration had commenced; and I have known either application to give prompt and effectual relief, even after the patient was suffering from severe rigors. A very popular remedy here, with both regular and irregular physicians, is a composition of kerosene and camphorated alcohol, applied as a liniment.—*Med. and Surg. Rep.*

**Obstinate Singultus Cured by Muriate of Pilocarpine.**—Dr. Ortille reports a case of persistent singultus, due to cerebral embolism, which proved utterly rebellious to all the usual methods of treatment. As the singultus persisted even during the sleep produced by morphine injections, and the strength of the patient was becoming greatly reduced, a hypodermic injection of half a grain of pilocarpine was at last administered. This produced abundant perspiration and salivation, and the hiccough ceased at once.—*All. Med. Cent. Zeil.*

**Paracotoin as a Remedy in Epidemic Cholera.** — Prof. Baelz, of Tokio, Japan, contributes to the Centralblatt of July 6th, an account of his striking success with this remedy in cases of malignant cholera. He administered it in doses of 0.2 gramme by hypodermic injection, suspended in equal parts of glycerine and water. The cure was prompt in five cases, all in which he used it, and the Japanese government has taken measures to provide a supply of the drug, in the event of another outbreak.

